# 

**Published:** 1912-06-15

**Authors:** 


### OUR BUREAU OF INFORMATION.

### Homes Offered and Wanted.

The columns of the Bureau this week afford evidence of the live spirit of charity which makes our voluntary institutions so varied and interesting a study. The range of homes which offer all the comforts and even elegancies of a refined life for the mere nominal weekly payment of 10s. a week is surprisingly large, and is barely known to the ordinary social worker. The impulse to help others, and especially those who have broken down in active work, is uncommonly strong among women who spend their lives in nursing, and for this small help in meeting outgoings many a generous mistress of an establishment will take a free patient, bestowing every bountiful care. Among inexpensive homes the Co-operative Sanatoria, under the direction of one of the Paddington Guardians and other well-known business men, are a most interesting experiment. In the fourth year of their existence the homes show a profit of £76, and all promises well for their continued success. There is room for a similar enterprise for female patients, the supply of inexpensive homes for epileptics being conspicuously below the demand. We are always glad to hear from the principals of any little homes offering accommodation in return for a small contribution towards maintenance, so that we may file the information for reference. In order to make this section of the Bureau of practical value to all engaged in social work there is need for a linking up of a great variety of homes offering accommodation at different rates, to the exclusion of such as are unsatisfactory in tone and management. More nursing homes with accommodation at moderate terms for accouchements are needed on the List.

### Rules for Correspondents.

1. Every letter, on whatever subject, must be accompanied by the coupon to be cut from the back cover (inside page) of The Hospital, current issue, and must contain the name and address of the correspondent with pseudonym for publication if desired.
2. Replies by post cannot be given, save under exceptional circumstances, at the Editor's discretion.
3. Letters from Approved Homes in reply to special needs published in the Bureau should state terms and full particulars, and be sent prepaid under cover to the Editor of the Bureau with coupon and name enclosed for identification.
4. Proprietors of homes which have not yet been entered on the List of Approved Homes, but have spare accommodation likely to suit special needs, are invited to write for an application form for registration. The fee for registration is 5s.
5. Special needs of every description relating to those who are sick or in difficulties are made known in these columns free of charge.
6. All communications to be addressed to The Editor of The Hospital Bureau of Information, 28 Southampton Street, Strand, London, W.C.

### Homes Wanted.

### For Infirm Man of 65.

"Troubled Daughter" (107a).—Your father might obtain admission to the St. Barnabas Home, 9 Lloyd Street, London, W.C. Write to the Sister-in-Charge. Another home for similar cases is at Torquay, and the charge is exactly what you name as being able to pay—viz., 10s. a week. Write to the Sister Superior, St. Raphael's Home, Torquay, from whom all particulars can be obtained. Write also to the Co-operative Sanatoria, Billericay, Essex, where possibly an arrangement might be made to meet you as regards terms since the patient does not appear to be helpless. Prepaid replies forwarded.

### For Maternity Case at Moderate Fees.

"M. E. G." (108a).—We note you want the address of a home where you can be received for your confinement at the end of August near London or Manchester, at moderate terms, and that you offer good references. We shall be pleased to forward letters to you from any of the Approved Homes on our List who write to you under the above initials. Other correspondents must please comply with Rule 4.

### Homes Offered.

### For Retired Nurse with very Light Duties.

Pretty suite of rooms offered for 10s. a week in small convalescent home near London to retired nurse willing to take an interest in the patients and give occasional help. Bracing situation in beautiful surroundings. Would suit nurse with small pension, as she would have good quarters and be quite free. H. O. B. [X 102].

### For Gentlewomen Needing Rest.

Home pleasantly situated near the sea at Brighton for the reception of six ladies not actually ill, but needing rest and change, which they are unable to afford themselves. A charge of 10s. a week is made towards maintenance. The invitation is for three weeks except by special arrangement. Mrs. R. [X 101].

Prepaid replies to either of the above sent under cover to the Editor of the Bureau will be forwarded.

### Approved Homes added to the List.

*Note.*—No home is added to the Approved List without direct medical testimony to its efficiency.

### Hydropathy and Massage.

Fulham Hydropathic Institute, 14, 16 Whittingstall Road, Munster Park, Fulham, S.W.—Principal, Miss Hannaford, trained mental nurse, assisted by masseur for male patients and by masseuse with certificates in massage, the Nauheim treatment, and medical electricity. Patients received under medical direction for treatment by the Weir Mitchell and also by the Mustard Pack Treatment. Owing to recent extension slight mental and medical cases can also be received at very moderate terms. Inspected and approved by Medical Officer of Health for district.

### Slight Mental or Epileptic Patients.

Co-operative Sanatoria, New Lodge and Leon House, Billericay, Essex.—Matron, Mrs. Edwards, cert. M.P.A. For the reception at very moderate charges of male patients suffering from slight mental ailments or epilepsy. Conducted on co-operative principles, the interest on share capital being limited to 5 per cent. Under medical direction. Fully qualified staff of nurses and attendants. Large grounds, with farm and recreation fields. Terms from 16s. to 42s. weekly, including laundry and all expenses.

### Institutions Abroad.

### Nursing Home in Naples.

Travel Editor, "Daily Mail."—There is an International Hospital at the Villa Bentinck, Naples, which is practically a nursing home. It is largely used by travellers of all nations, and has accommodation for private patients. The medical director is Dr. Scotti, who speaks English.

### Appointments.

Mr. W. J. Dakin, D.Sc., at present assistant lecturer and demonstrator in zoology in the University of Liverpool, has been appointed senior assistant in the Department of Zoology and Comparative Anatomy at University College, London.

Dr. Eric Bellingham Smith, M.D., M.R.C.P., has been appointed physician to out-patients at the Great Northern Central Hospital, Holloway Road, N.

Mr. Raymond B. Etherington-Smith, M.A., M.B., S.C., F.R.C.S., and Mr. William Ernest Miles, F.R.C.S., M.R.C.S., L.R.C.P., have been appointed honorary consulting surgeons to the Royal Hospital, Richmond, Surrey.

### Gifts and Bequests.

The Samaritan Free Hospital for Women has received £100 from Smith's (Kensington Estate) Charity.

The Trustees of Smith's (Kensington Estate) Charity have sent a donation of £200 to Queen Charlotte's Hospital.

The Chelsea Hospital for Women has received from the Trustees of Smith's (Kensington Estate) Charity £250 for its Rebuilding Fund.

The Florence Nightingale Hospital for Gentlewomen, 19 Lisson Grove, has received £21 from the Merchant Taylors' Company and £10 10s. from the Skinners' Company.

Mr. Peter Campbell, Perth, has left £200 to the Baptist Medical Mission, and £100 each to the County and City Royal Infirmary, Perth, and the Society for the Relief of Incurables in Perth and Perthshire.

The Royal Dental Hospital, Leicester Square, has received a donation of 25 guineas from the Mercers' Company and one of ten guineas from the Skinners' Company, also an annual subscription of ten guineas from A. M. Singer, Esq.

A Bradford solicitor, the late Mr. James Norton Dickons, who left a net estate of £30,262, has bequeathed the residue of his property, which will amount to about £20,000, in equal shares to the Bradford Infirmary, the Royal Halifax Infirmary, and three other institutions.

Miss Elizabeth Helen Nash, of Bristol, has left £50 to the Bristol Blind Asylum.

The King has sent his annual subscription of fifteen guineas to the Royal London Ophthalmic Hospital (Moorfields Eye Hospital).

Mrs. Emily Cadwalladr, of Swansea, left £1,000 to the Swansea Hospital for the endowment of a bed and a further £1,000 for the Extension Fund.

Mrs. Catherine Elizabeth Wilkin, Powys, Sidmouth, Devon, has left £500 each to Sidmouth Cottage Hospital, Hayes Hospital, and the Home for Incurables, Putney.

The Rev. Charles Frederick Gore, of Mountjoy, Brighton, for many years vicar of Edenbridge, Kent, who left estate of the gross value of £109,781, has bequeathed £200 each to the Tunbridge Wells General Hospital and the Sussex County Hospital.

Mr. James Brown, of Newcastle, left £1,000 each to the Royal Victoria Infirmary, Newcastle, and the Berwick-upon-Tweed Infirmary; £400 to the Fleming Memorial Hospital for Sick Children; and £300 to the Newcastle-upon-Tyne Maternity Hospital.

The Leicester Infirmary has received the following legacies:—£250 to the infirmary and £200 to the children's hospital from the late Rev. Henry Homer, and £20 from the late Mr. Mark Foxon. Moreover, by the will of the late Mr. J. S. Cooper, of Bournemouth, a fourth share of the residuary estate is bequeathed to the infirmary.

Mr. Thomas Yates, of Liverpool, has left £1,000 to the Liverpool Infirmary for Children, and £500 each to the Liverpool Bluecoat Hospital, the Children's Convalescent Home, West Kirby, Cheshire, the Liverpool Medical Missionary Society, the Liverpool Convalescent Homes, Woolton, and the Hospital for Consumption and Diseases of the Chest, Liverpool.

A Munificent Bequest.—Gourock, the popular Clyde resort in the West of Scotland, has benefited by the death of a well-known figure, Captain Duncan McPherson; he bequeathed the sum of £17,000 for the erection and endowment of a cottage hospital, which will fill a long-felt want.

### The Week's Novelties.

### THE "SANITAS" COMPANY, LIMITED.

Presiding over the recent annual general meeting of the "Sanitas" Company, the Chairman, Mr. C. T. Kingzett, remarked that two directions in which good progress had been made were the increased use of "Sanitas Fluid" as a fob for preventing the bites of mosquitoes, gnats, and other insects, and that of "Sanitas Powder" for ridding gardens of slugs. All products manufactured by the "Sanitas" Company are tested in their own laboratories before sale.

### THE MALVERN WATER.

The reputation of the Malvern water on the score of natural purity has always been a high one, and it is well to know that this palatable spring-water can be conveniently procured in bottles from Messrs. W. and J. Burrow, Great Malvern. The natural Malvern is a still water, but it is also to be had aerated if a pure effervescent table-water is desired. Particulars as to the cost of these waters, their free delivery, etc., may be obtained from the proprietors of the spring at the above address. No Continental waters surpass the Malvern in purity, and the latter not being medicinal may be drunk without fear of producing a lowering of the vitality, but on the other hand their refreshing properties are great.

